# Identification of immunotherapy-related lncRNA signature for predicting prognosis, immunotherapy responses and drug candidates in bladder cancer

**DOI:** 10.1186/s12885-023-10828-z

**Published:** 2023-04-18

**Authors:** Pengyu Hui, Feng Ni, Liang Zheng, Lei Jia, Zhe Wang

**Affiliations:** 1 Department of Urology, The Second Affiliated Hospital of Xi’an Medical University, No.167 Fangzhicheng East Road, Baqiao District, Xi’an, Shaanxi 710038 China; 2 Department of Urology, Cancer Hospital of China Medical University, No.44 Xiaoheyan Road, Dadong District, Shenyang, 110042 Liaoning China; 3 Department of Urology, Liaoning Cancer Hospital & Institute, No.44 Xiaoheyan Road, Dadong District, Shenyang, 110042 Liaoning China

**Keywords:** Bladder cancer, Immunotherapy, Long non-coding RNA, Prognostic, ceRNA network, Molecular docking

## Abstract

**Background:**

Bladder cancer (BC) is one of the most common malignant diseases and the most common causes of cancer death worldwide. Immunotherapy has opened new avenues for precision treatment of bladder tumours, and immune checkpoint inhibitors (ICIs) have revolutionized the clinical treatment strategy of bladder tumours. In addition, long non-coding RNA (lncRNA) plays an important role in regulating tumour development and immunotherapy efficacy.

**Methods:**

We obtained genes with significant differences between anti-PD-L1 response and non-response from the Imvogor210 data set and combined with the bladder cancer expression data in the TCGA cohort to obtain immunotherapy-related lncRNA. Based on these lncRNAs, the prognostic risk model of bladder cancer was constructed and verified by GEO external data set. The characterization of immune cell infiltration and immunotherapy effects between high-risk and low-risk groups were then analysed. We predicted the ceRNA network and performed molecular docking of key target proteins. The functional experiments verified the function of SBF2-AS1.

**Results:**

Three immunotherapy-related lncRNAs were identified as independent prognostic biomarkers for bladder cancer and a prognostic model of immunotherapy-related prognosis was constructed. Prognosis, immune cell infiltration, and immunotherapy efficacy were significantly different between high- and low-risk groups based on risk scores. Additionally, we established a ceRNA network of lncRNA(SBF2-AS1)-miRNA(has-miR-582-5p)-mRNA (HNRNPA2B1). Targeting the protein HNRNPA2B1 identified the top eight small molecule drugs with the highest affinity.

**Conclusion:**

We developed a prognostic risk score model based on immune-therapy-related lncRNA, which was subsequently determined to be significantly associated with immune cell infiltration and immunotherapy response. This study not only helps to promote our understanding of immunotherapy-related lncRNA in the prognosis of BC, but also provides new ideas for clinical immunotherapy and the development of novel therapeutic drugs for patients.

**Supplementary Information:**

The online version contains supplementary material available at 10.1186/s12885-023-10828-z.

## Background

Bladder cancer (BC) is the second most common malignant tumor in the urinary system, and the most common pathological type is urothelial cell carcinoma, accounting for more than 90% of all bladder cancers [1]. 70–80% of bladder cancer patients are diagnosed with non-muscle invasive bladder cancer (NMIBC), and most of them will relapse after treatment, making bladder cancer a high tendency of recurrence in pan-cancer [2]. However, there is a lack of reliable serum biomarkers for early diagnosis and prediction of clinical progress of bladder cancer. Therefore, there is an urgent need for a series of new independent biomarkers based on comprehensive genomic analysis to better predict the clinical outcome of patients with bladder cancer.

Immunotherapy has opened up a new way for the precise treatment of malignant tumors. in the past decade, immune checkpoint inhibitors (ICI) have completely changed the clinical treatment pattern of many advanced malignant tumors, and greatly reversed the current situation of relying on surgery, radiotherapy, chemotherapy and hormone therapy [3]. Programmed cell death receptor 1 (PD-1) is expressed on tumor infiltrating lymphocytes (TILs) and regulates T cell activation and response. it is a key immune checkpoint receptor in tumor-induced immunosuppression.

It has been proved that targeted blocking of the relationship between PD-1 and its ligand PD-L1 on tumor cells can enhance the anti-tumor activity of effector T cells and alleviate the patient's condition [4, 5].

Based on the results of the new generation sequencing, only about 2 base pairs encode proteins in the human genome, and most genomes are non-coding protein sequences, including repetitive regions, gene introns, non-coding RNA and intergenic regions [6]. At present, most studies on base pairs of non-coding proteins are focused on non-coding RNA. According to the size of non-coding RNA, it is subdivided into small non-coding RNA (< 200nt) and long chain non-coding RNA (lncRNA; > 200nt) [7]. LncRNA plays a key role in many biological processes, including cell proliferation, differentiation, metabolism and diseases, including cancer [8]. In terms of mechanism, lncRNA as a regulator participates in tumorigenesis and development through a variety of mechanisms, such as protein and RNA scaffolds, competitive endogenous RNA (ceRNA), transcriptional or post-transcriptional regulation and epigenetic modification [9].

In this study, we identified immunotherapy-related lncRNA in bladder cancer using immunotherapy data sets. We also established and verified a model based on immunotherapy-related lncRNA to predict the prognosis of patients with bladder cancer, and showed a good predictive ability. We further assessed the differences in immune microenvironment, chemotherapeutic drug sensitivity and immunotherapy response between high-risk and low-risk groups. Then we build a ceRNA network based on SBF2-AS1 and find small molecular drugs that can target HNRNPA2B1 through molecular docking. Finally, we did in vitro experiments to verify the function of SBF2-AS1.

## Materials and methods

### Data source

We downloaded clinical data, miRNA expression data, lncRNA expression data, and somatic mutation data from the Cancer Genome Atlas (TCGA) database (https://portal.gdc.cancer.gov/) [10]. All raw RNA-seq and miRNA-seq data are normalized to fragments of one millionth of a thousand bases (FPKM). We downloaded the expression data and clinical features from the GEO database as external data sets for verification. We also downloaded the IMvigor210 dataset, a set of expression data and clinical information from patients with urothelial cancer treated with atezolizumab (PD-L1 blocker) [11].

### Identify genes that differ in response to immunotherapy

We set the IMvigor210 dataset CR and PR as the response group to PD-L1 blockers, and SD and PD as non-response groups to PD-L1 blockers. The differential expression of lncRNA and mRNA in immunotherapy was identified by R packet "DESeq2" (bioconductor.org/packages/devel/bioc/html/DESeq2.html) by comparing the response group and non-response group with threshold value (FDR < 0.05, | log Fc |> 0.75). The R package "ggplot2" (https://www.rdocumentation.org/packages/ggplot2/) is then used to visualize the volcano map and the Wayne diagram.

### Construction of prognostic models

The best prognostic risk model was established by univariate Cox and multivariate Cox regression analysis. Risk score per patient for bladder cancer: risk score = Coef(TFAP2A-AS1) × Expr(TFAP2A-AS1) + Coef(SBF2-AS1) × Expr(SBF2-AS1) + Coef(RRN3P2) × Expr(RRN3P2). Expr represents the expression level of a particular gene, and Coef is obtained by univariate cox regression and then multivariate cox regression analysis, which represents the coefficient of gene Cox analysis in the model.

### CeRNA network prediction

We used the LncATLAS database (https://lncatlas.crg.eu/) to identify subcellular localization of lncRNA [12]. The ceRNA network of lncRNA and the LncACTdb database (http://bio-bigdata.hrbmu.edu.cn/LncACTdb/) and ENCORI database (https://starbase.sysu.edu.cn/) were predicted. where the threshold for the ENCORI database is set to high stringency [13, 14].

### Immune microenvironment analysis

Tumor purity, matrix score, immune score and ESTIMATE score were calculated by R package "ESTIMATE" [15]. Single sample gene set enrichment analysis (ssGSEA) algorithm studies the level of immune infiltration between high-risk and low-risk groups based on different immune cell types and immune functions. We also evaluated the association between HNRNPA2B1 and immunoinfiltrating cells, including B cells, CD8 + T cells, CD4 + T cells, macrophages, neutrophils, and dendritic cells, in the Tumor Immune Evaluation Resource (TIMER, http://cistrome.shinyapps.io/timer/).

### Predicts immunotherapy response

The Tumor Immune Dysfunction and Exclusion (TIDE) algorithm can be used to infer the patient's effect on immunotherapy [16]. TIDE scores were inversely correlated with immunotherapy efficacy. Download the IPS scores for bladder cancer anti-PD-1 and anti-CTLA4 from the TCIA database (https://tcia.at/home) to evaluate the association between risk scores based on immunotherapy-related lncRNA construction and the efficacy of PD-1 and CTLA4 blockers [17].

### Molecular docking

We use MOE software to simulate molecular docking of target proteins and small molecule inhibitors. The three-dimensional structure of the target protein is downloaded from the PDB database, and the small molecule inhibitors are FDA-approved drugs, downloaded from the zinc15 database and converted into three-dimensional structures in the MOE software. We optimize proteins, such as removing water molecules and ligands and replenishing hydrogen atoms and protons, and minimizing energy for small molecule inhibitors. Finally, the binding mode of HNRNPA2B1 with small molecule drugs was studied by docking simulation.

### Cell culture and small interfering RNA (siRNA) transfection

We used bladder cancer cell lines T24 and UC3, purchased from the Chinese Academy of Sciences Cell Bank. T24 and UC3 cells were cultured in RPMI-10 medium (Procell) supplemented with 1640% fetal bovine serum. siRNA purchased from JTSBIO Co. (China) was transfected with lipo3000. The bladder cancer cells were spread on the six-well plate 24 h before transfection, and transfection was carried out when the cell density grew to about 70%. First, 7.5 μ L siRNA and 125 μ L serum-free medium were mixed and incubated at room temperature for 5 min, and 8 μ L Lipo3000 and 125 μ L serum-free medium were incubated at room temperature for 5 min. The above two solutions were then mixed and incubated for 15 min. Finally, the mixture was added to the six-well plate, and the transfection efficiency was analyzed after 48 h. The siRNA sequence is as follows: GATCCAGATGGAGGAAACATCTCTGAT.

### CCK8 assay

CCK8 is used to detect cell viability. After the cells were digested and washed and resuscitated, the cells were counted by cell counting board, and the si-SBF2-AS1-transfected T24 and UC3 cells were inoculated in a 96-well plate, then 2000 cells were inoculated into each well, with 6 multiple holes in each group, and 24 h, 48 h and 72 h groups. Cell counting kit-8 (CCK8) was added to each well at 24 h, 48 h and 72 h, respectively, and then the absorbance of each well was measured at 450 nm using multimode enzyme labeling instrument. Each experiment was repeated three times.

### Clonal formation assay

The cells were digested, cleaned and resuscitated, and then counted by cell counting board. About 2000 normal control and si-transfected T24 and UC3 cells were inoculated in petri dish and shook until the cells were evenly distributed in the dish. The cells were cultured in cell incubator for 2 weeks, during which the cell clone formation was observed regularly and the medium was changed after 1 week of culture. When the shape and size of the cell clone is appropriate, discard the culture medium, add PBS and wash for three times, then add 4% paraformaldehyde and fix 30 min at room temperature. The paraformaldehyde was discarded and washed with PBS for three times. Under the condition of avoiding light, the colony was stained with 1 ml 0.1% crystal violet for 30 min. Discard crystal violet, wash with PBS for three times, and then observe and take pictures under microscope after air-drying.

### Assay of cell invasion ability

Transwell test was used to determine the invasive ability of cells. The transfected T24 and UC3 cells were re-suspended in serum-free RPMI-10 medium and cultured on the surface of the upper chamber coated with matrix glue. After 24 h, the cells adhered to the lower membrane were fixed with 4% paraformaldehyde and stained with crystal violet.

### Statistical analysis

All statistical analyses were performed in R (http://www.r-project.org/). Two and more groups were compared between groups using Wilcoxon's test and Kruskal–Wallis test, respectively. Correlation was assessed by Spearman correlation analysis. Statistical analysis was performed using two-tailed unpaired t test, one-way analysis of variance, and two-way analysis of variance using GraphPad Prism software. A p-value ≤ 0.05 was considered statistically significant.

## Results

### Risk scores were constructed based on immunotherapy-related lncRNA

**Fig. 1:**
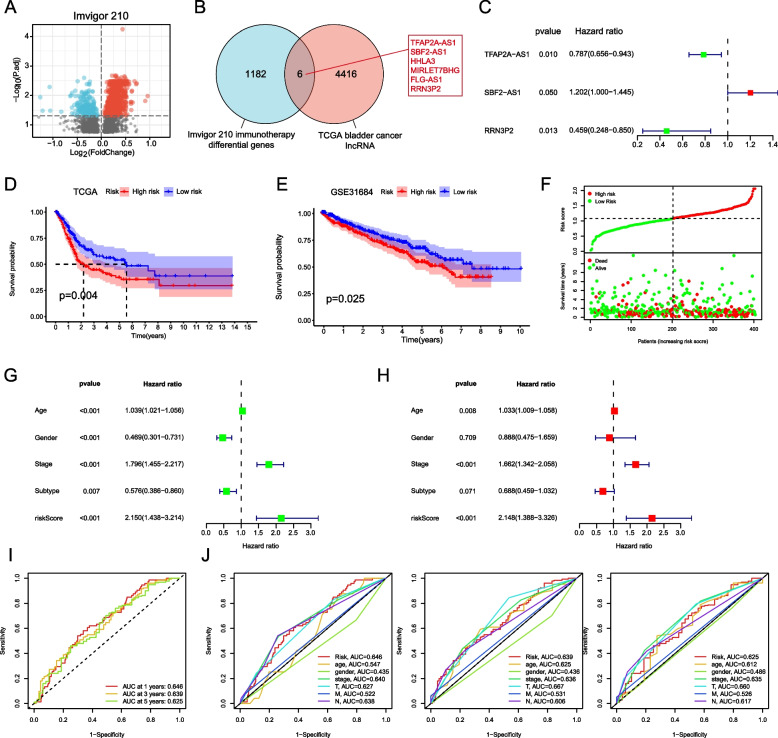
Construction of prognostic risk model. **A** Volcano map of immunotherapy-differential lncRNA in the Imvigor210 dataset. **B** Intersection Venn diagram of immunotherapy differential lncRNA and TCGA lncRNA for bladder cancer. **C** Forest maps of three lncRNAs were constructed. **D** Survival curves between high-risk and low-risk groups in the TCGA dataset. **E** Survival curves between high-risk and low-risk groups in the GSE31684 dataset. **F** The relationship between risk score and patient survival status. Univariate **G** and multivariate (**H**) regression forest plots for each clinical trait and risk score. **I** ROC curves of bladder cancer patients at 1, 3 and 5 years. **J** ROC curves constructed by risk scores and clinical traits at the 1st, 3rd and 5th years

We first screened out genes with significant differences between responses to PD-L1 blockers and non-responses through Imvigor210 data sets (Fig. 1A). Then, we intersected the above genes with lncRNA in TCGA bladder cancer to obtain 6 lncRNAs. They are TFAP2A-AS1, SBF2-AS1, HHLA3, MIRLET7BHG, FLG-AS1 and RRN3P2 respectively (Fig. 1B). We analyzed the difference and prognosis of immune-related lncRNA, followed by multivariate Cox regression analysis. Results of univariate and multivariate cox analysis of 6 immunotherapy-related lncRNAs (Table S1). Finally identified three immunotherapy-related lncRNAs to build risk models, including TFAP2A-AS1, SBF2-AS1 and RRN3P2 (Fig. 1C). According to the median risk score, bladder cancer patients in the TCGA cohort were divided into low-risk group and high-risk group. The Kaplan–Meier survival curve showed that the survival rate of the high risk group was significantly lower than that of the low risk group. (Fig. 1D). We used GSE31684 as an external data set to verify the prognostic characteristics of the model, and the survival curve showed that the prognosis was poor in the high risk group (Fig. 1E). Analysis of the correlation between risk score and survival state heat map showed that the higher the risk score, the higher the mortality of patients (Fig. 1F). Through univariate and multivariate regression analysis of forest map results, risk score was an independent risk factor (Figs. 1G, H). We used the time-dependent and clinical characteristics of the receiver operating characteristics (ROC) to evaluate the sensitivity of the model to prognosis, and evaluated the results according to the area under the ROC curve (AUC). The results showed that ROC was more accurate in predicting the prognosis of BC patients (Fig. 1I, J).

### Nomogram based on immunotherapy-related lncRNA risk scores

**Fig. 2:**
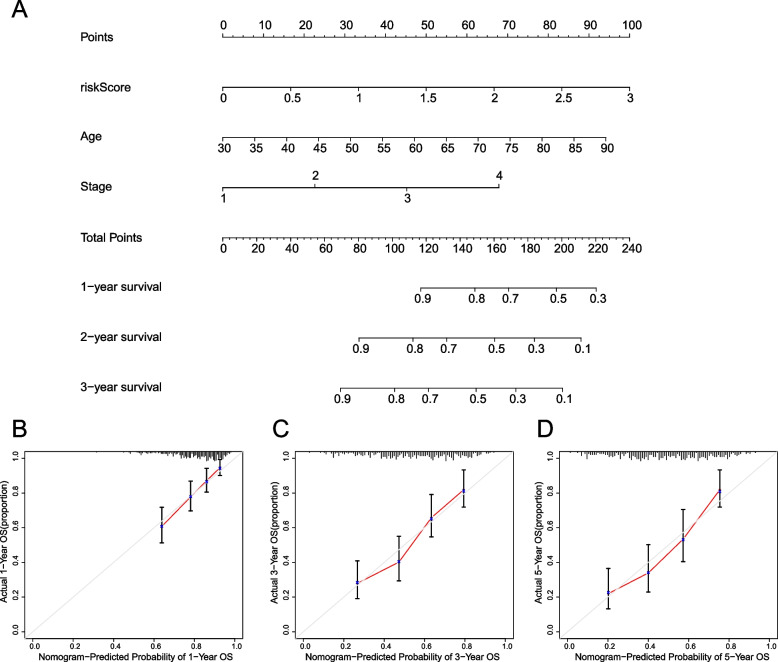
Nomogram based on risk score. **A** Nomogram constructed based on age, clinical stage, and risk score. The calibration curve shows the agreement between predicted survival and actual survival at 1 year (**B**), 3 years (**C**), and 5 years (**D**) based on bias-adjusted prognostic nomograms

The results of univariate and multivariate regression analysis showed that age, clinical stage and risk score were independent risk factors and could independently predict the survival rate of patients. Therefore, we construct a line map based on age, clinical stage and risk score, in which risk score is the main component of the total score (Fig. 2A). We further constructed a calibration curve for the diagram, and the results showed that the predicted survival rates of patients in the 1st, 3rd and 5th years were consistent with the actual survival rates (Fig. 2B-D).

### Mutation status in different risk groups

**Fig. 3:**
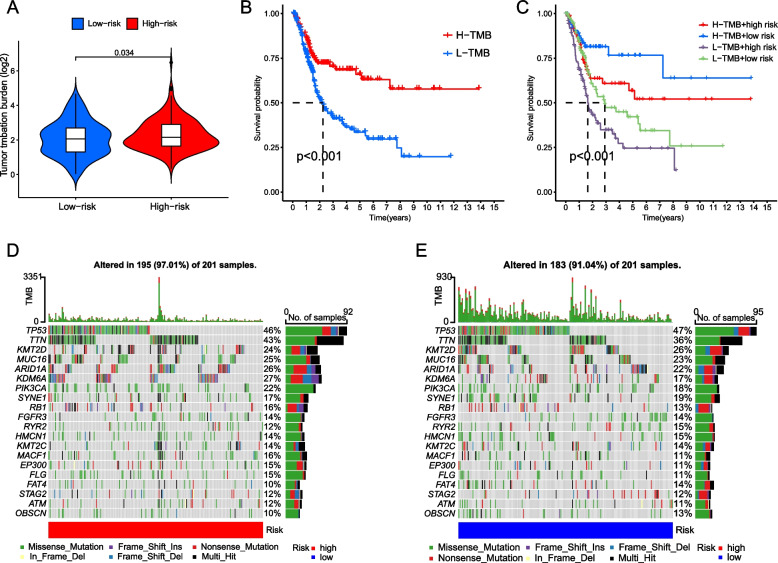
Analysis of tumor mutation burden in high-risk group and low-risk group. **A** Box plot of the difference in TMB between the high-risk group and the low-risk group. **B** Survival curves of high-risk and low-risk groups. **C** Risk score compared with survival curve of bladder cancer patients with TMB combined stratified by stratified risk. The waterfall chart shows the frequency of mutations in the top 20 genes in the high-risk group (**D**) and low-risk group (**E**)

Tumor mutation load (TMB) plays an important role in clinical practice, and we further study the difference of TMB between high-risk group and low-risk group. The results showed that the TMB of high risk group was significantly higher than that of low risk group (Fig. 3A). Survival analysis showed that patients with high TMB had more significant survival advantages than patients with low TMB (Fig. 3B). When the risk score was integrated with TMB, the bladder cancer patients in the TCGA cohort were divided into four groups. The high TMB and low risk groups had the most significant survival advantage, while the low TMB and high risk groups showed poor prognosis (Fig. 3C). In addition, we also showed the mutation frequency of the first 20 genes in different risk groups of bladder cancer by waterfall map (Fig. 3D, E).

### Tumor microenvironment and immunotherapy analysis

**Fig. 4:**
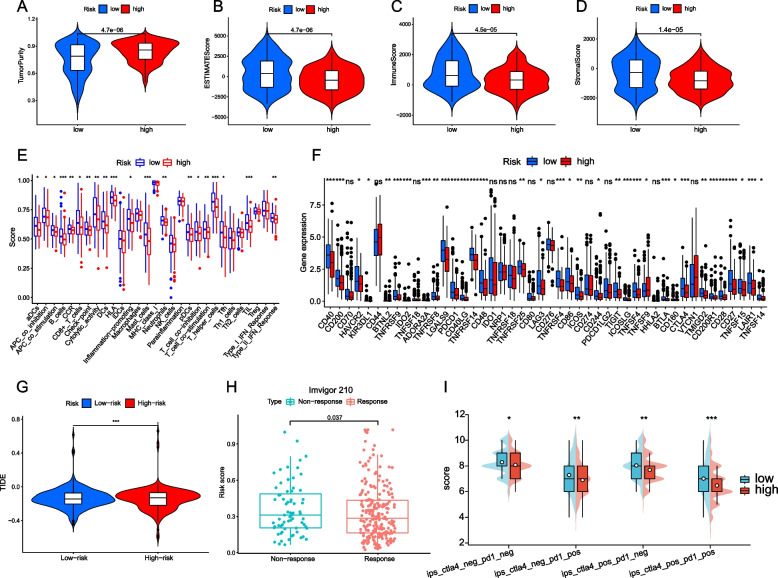
TME characterization and immunotherapy analysis between high-risk and low-risk groups. Differences between high-risk and low-risk groups in tumor purity (**A**), ESTIMATE Score (**B**), immunological score (**C**), and matrix score (**D**). **E** Differences in immune infiltration cells and immune function between the high-risk group and the low-risk group. **F** Differences in population immunity checkpoints between high-risk and low-risk groups. **G** Differences in TIDE between high-risk and low-risk groups. **H** Differences in risk scores between the anti-PD-L1 immunotherapy response group and the non-response group in the Imvigor210 cohort. **I** Differences in CTLA4(-) and PD-1(-), CTLA4(-) and PD-1( +), CTLA4( +) and PD-1(-),CTLA4( +) and PD-1( +) between high-risk and low-risk groups

Tumor microenvironment (TME) is not only a complex assembly of tumor cells, immune infiltrating cells, stromal cells and extracellular components, but also a key factor in tumor-immune interaction, affecting tumor response to immunotherapy [18]. We analyzed the difference of TME composition between the high risk group and the low risk group. The results showed that the tumor purity of the high risk group was significantly higher than that of the low risk group, while the ESTIMATEScore, immune score and matrix score of the low risk group were significantly lower than those of the high risk group (Fig. 4A-D). We further analyzed the differences of immune infiltrating cells and immune function between the two groups. Most of the immune infiltrating cells and immune function were significantly decreased in the low risk group, such as DCs, B cells, CD8 + T cells, T helper cells and immune checkpoints. (Fig. 4E). After that, we analyzed the immune checkpoint, and compared with the patients in the high-risk group, the low-risk group showed a higher level of immune checkpoint expression, suggesting the difference in the therapeutic effect of immune checkpoint inhibitor (ICI) between the two groups (Fig. 4F).

We then further studied the correlation between the risk score and the therapeutic effect of ICI. First, by calculating the TIDE score of each patient, the results showed that the patients in the low-risk group had a lower TIDE score, suggesting that the patients in the low-risk group had a better effect on immunotherapy (Fig. 4G). After verifying the correlation between immunotherapy and risk score on Imvigor210 dataset, we found that the risk score of patients who responded to PD-L1 blockers was significantly lower than that of non-response groups, that is, patients with low risk had better efficacy on PD-L1 blockers (Fig. 4H). We also evaluated the response of bladder cancer patients to PD-1 and CTLA4 blockers by IPS score. The results showed that IPS was higher in patients with low risk, suggesting that our low risk group was more effective in treating single or combined blockers of PD-1 and CTLA4 (Fig. 4I).

### Drug susceptibility analysis

**Fig. 5:**
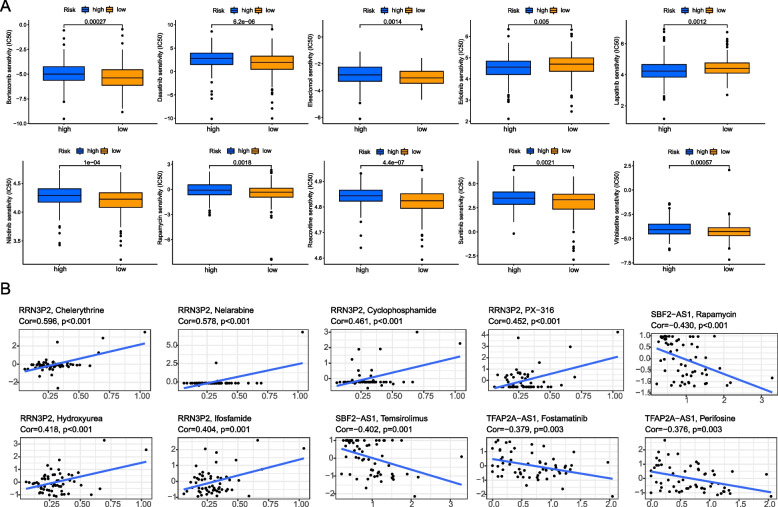
Drug susceptibility analysis. **A** Sensitivity of chemotherapy drugs between high-risk and low-risk groups. **B** RRN3P2, SBF2-AS1 and TFAP2A-AS1 gene expression correlated with drug susceptibility

To explore the potential use of drugs in bladder cancer chemotherapy, we evaluated the semi-maximum inhibitory concentration (IC50) of chemotherapeutic drugs between the high-risk group and the low-risk group. The results showed that the IC50 values of Erlotinib and Lapatinib in the high risk group were lower, suggesting that the patients in the high risk group may benefit more from Erlotinib and Lapatinib. The low risk group has lower IC50 values for Bortezomin, Dasatinib, Elesclomol, Nilotinib, Rapamycin, Roscovitine, Sunitinib and Vinblastine, which means that the low risk group may benefit more from the above chemotherapeutic drugs (Fig. 5A). Using cellMiner database to analyze the correlation between genes and drug sensitivity, we found that RRN3P2 was positively correlated with most drug sensitivity, that is, the stronger the expression of RRN3P2, the more sensitive patients were to these drugs (Fig. 5B) [19].

### Construction and validation of ceRNA networks based on immunotherapy-related lncRNA and mRNA

**Fig. 6:**
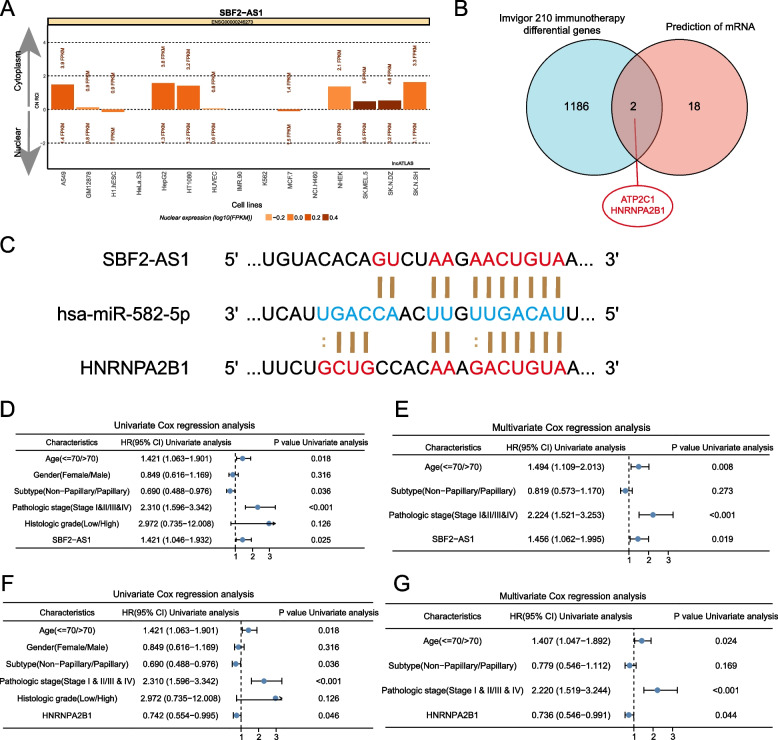
Construction of the ceRNA network. **A** Prediction of cell localization of SBF2-AS1 by lncATLAS. **B** The Wenn diagram obtained by the intersection of the mRNA regulated by SBF2-AS1 in the LncACTdb and ENCORI databases and the immunotherapy differential genes in the Imvigor210 dataset. **C** Base pairing between has-miR-582-5p and SBF2-AS1 and HNRNPA2B1. Univariate (**D**) and multivariate (**E**) Cox regression analysis of SBF2-AS1 in bladder cancer. Univariate (**F**) and multivariate (**G**) Cox regression analysis of HNRNPA2B1 in bladder cancer

According to the theory of ceRNA network mechanism, lncRNA can regulate mRNA expression by interacting with miRNA. Through lncATLAS localization analysis of immunotherapy-related lncRNA cells, it was found that SBF2-AS1 was mainly located in the cytoplasm and could be used as ceRNA to regulate mRNA expression through spongification (Fig. 6A). We further predicted the mRNA and miRNA regulated by SBF2-AS1 through LncACTdb and ENCORI databases, and intersected the predicted mRNA and Imvigor210 datasets of immunotherapy differential genes to get two mRNAs, namely ATP2C1 and HNRNPA2B1 (Fig. 6B). After that, we further increased the predicted threshold, found that the correlation of SBF2-AS1/has-miR-582-5p/HNRNPA2B1 signaling pathway was the highest, and predicted the base pairing of has-miR-582-5p associated with SBF2-AS1 and HNRNPA2B1 (Fig. 6C).

We further studied the prognostic significance of the expression of SBF2-AS1 and HNRNPA2B1, and determined the characteristics of OS by univariate and multivariate Cox regression analysis.

In the TCGA cohort of bladder cancer patients, the expression of SBF2-AS1 and HNRNPA2B1 is significantly correlated with the prognosis, and may be an independent prognostic factor in patients with Bladder cancer (Fig. 6D-G). In addition, the prognostic significance of hsa-miR-582-5p in bladder cancer was analyzed by COX regression and Kaplan–Meier curve, and it was found that it had no significant significance in the prognosis of bladder cancer (Table S2 and Figure S1A). The expression of SBF2-AS1 and hnRNPA2B1 in bladder cancer cells was significantly higher than that in normal tissues, but there was no significant difference in hsa-miR-582-5p between normal bladder tissues and bladder cancer cells (Figure S1B-C).

### Association of HNRNPA2B1 between TME and immunotherapy

**Fig. 7:**
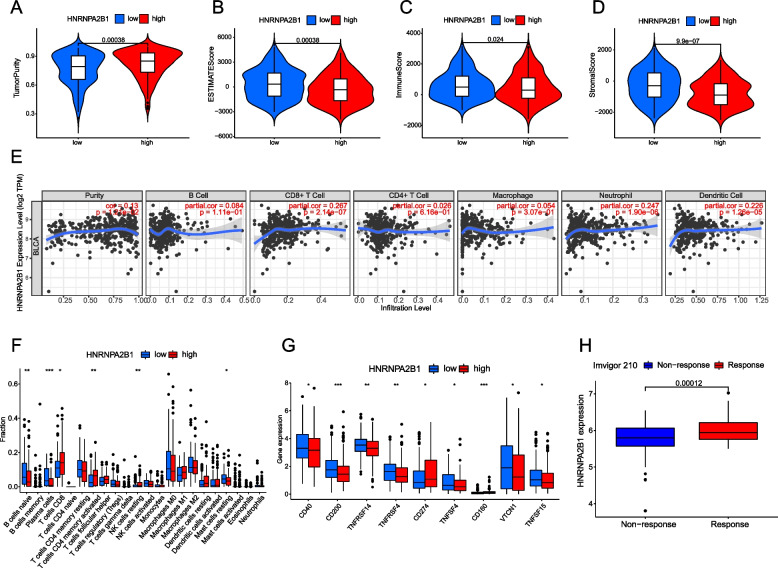
Association between HNRNPA2B1 and Immune-infiltrating cells. Differences between tumor purity (**A**), ESTIMATEScore (**B**), immunoscore (**C**), and matrix score (**D**) in the HNRNPA2B1 high- and low-expression groups. **E** Correlation between HNRNPA2B1 expression and immune cell invasion in bladder cancer. Differences between immunoinfiltrating cells (**F**) and common immune checkpoints (**G**) in groups with high and low expression of HNRNPA2B1. **H** Differences in HNRNPA2B1 expression between the response and non-response groups in the Imvigor210 immunotherapy dataset

We analyzed the difference of TME composition between HNRNPA2B1 high expression group and low expression group. The results showed that the tumor purity of high expression group was significantly higher than that of low expression group, while the ESTIMATEScore, immune score and matrix score of low expression group were significantly higher than those of high expression group (Fig. 7A-D). We then evaluated the relationship between HNRNPA2B1 expression and immune infiltration of bladder cancer, and found that HNRNPA2B1 expression was positively correlated with the infiltration of CD8 + T cells, neutrophils and dendritic cells (Fig. 7E). The difference analysis of immune cell infiltration between HNRNPA2B1 high expression group and low expression group showed that CD8 + T cells in HNRNPA2B1 high expression group were significantly higher than those in low expression group (Fig. 7F). We also analyzed the difference of common immune checkpoints between HNRNPA2B1 high expression group and low expression group. Compared with HNRNPA2B1 low expression group, CD274 (PD-L1) increased significantly in HNRNPA2B1 high expression group (Fig. 7G). Finally, we observed in the Imvigor210 immunotherapy data set that the expression of HNRNPA2B1 in the response group was significantly higher than that in the non-response group to PD-L1 blockers (Fig. 7H). There was significant difference in SBF2-AS1 between anti-PD-L1-responsive group and non-reactive group (*p* < 0.05), and the expression level of SBF2-AS1 in anti-PD-L1-responsive group was significantly higher than that in non-reactive group (Figure S1D). By analyzing the relationship between SBF2-AS1, has-miR-582-5p and TME, we found that there was no difference in immune cell infiltration among SBF2-AS1 differential expression groups, but the level of immune infiltration in low has-miR-582-5p expression group was significantly higher than that in high has-miR-582-5p group (Figure S1E-I).

### Molecular docking posture of HNRNPA2B1 and small molecule drugs

According to previous studies, targeted HNRNPA2B1 can reduce drug resistance of cancer cells to endocrine therapy [20]. Therefore, we docked HNRNPA2B1 with small molecules to find small molecules that can target HNRNPA2B1.

**Fig. 8:**
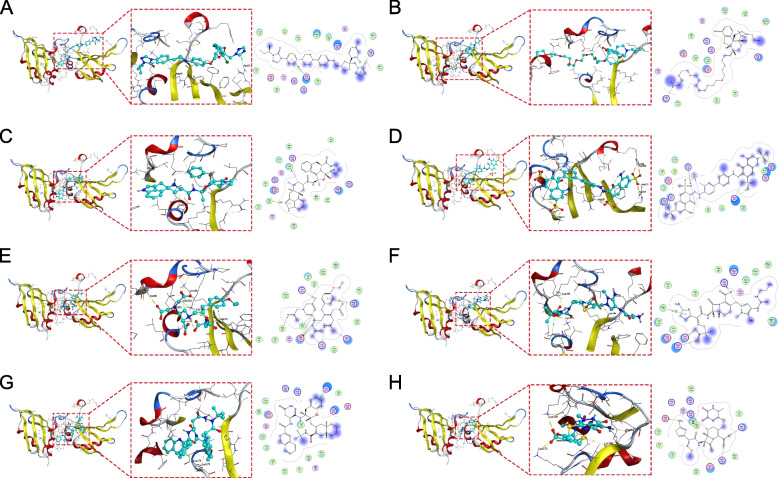
Screening of drug candidates targeting HNRNPA2B1. Molecular docking posture of HNRNPA2B1 active pocket with Itc (**A**), Naloxegol (**B**), Dihydroergotamine (**C**), Trypan Blue (**D**), Eovist (**E**), Ceftolozane (**F**), Saquinavir (Sqv) (**G**) and Ceftriaxone (**H**). On the left is the overall structure diagram of the target protein and small molecule drug. In the middle is a detailed diagram of the interaction between the target protein and the small molecule drug, in which the hydrogen bond is shown with a yellow dotted line and the H-pi bond is shown with a green dotted line. On the right is the 2D interaction diagram between the target protein and the small molecule drug, the green arrow indicates that the small molecule interacts with the HNRNPA2B1 sidechain hydrogen bond, the blue arrow indicates that the small molecule interacts with the HNRNPA2B1 backbone chain hydrogen bond, and the hexagon represents the interaction of HNRNPA2B1 amino acid residues with aromatic hydrocarbons

We use MOE software to simulate the binding posture of HNRNPA2B1 to small molecular drugs, in which the interactions between target proteins and small molecules are summarized in Table S3, and show the first eight small molecular drugs with the strongest binding to HNRNPA2B1, including Itc, Naloxegol, Dihydroergotamine, Trypan Blue, Eovist, Ceftolozane, Sqv and Ceftriaxone (Figs. 8A-H). For example, Trypan Blue (ZINC000169289767) forms hydrogen bond and ion bond interaction with HNRNPA2B1 amino acid residues Lys-173, Lys104, Lys186, Gly-106, Arg-185, Arg-190 and Met-193, in which Gly106 is used as hydrogen bond receptor and Lys-173, Lys104, Lys186, Arg-185, Arg-190 and Met-193 as hydrogen bond donors. Ceftriaxone (ZINC000028467879) forms hydrogen bond and ionic bond interaction with HNRNPA2B1 amino acid residues Lys-186, Lys-173, Asp-167 and Lys104, in which Asp-167 is used as hydrogen bond acceptor and Lys-186, Lys-173 and Lys-104 as hydrogen bond donors.

### SBF2-AS1 knockdown inhibited the proliferation and migration of bladder cancer cells

**Fig. 9:**
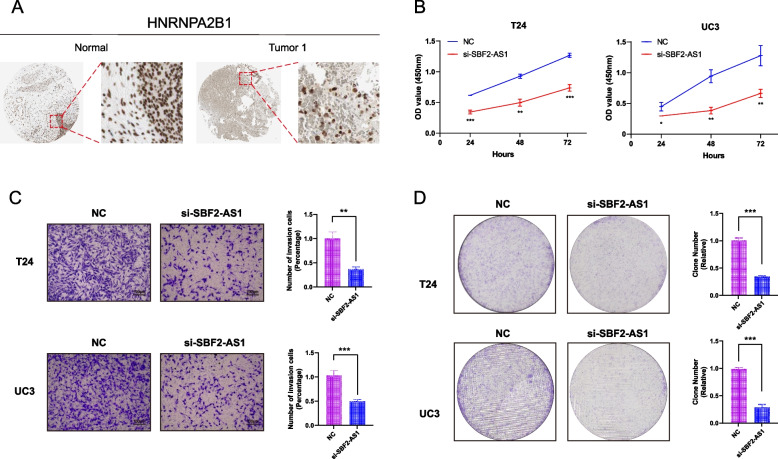
HNRNPA2B1 protein expression level and SBF2-AS1 knockdown inhibited bladder cancer cell proliferation and migration. **A** Protein expression levels of HNRNPA2B1 in normal and tumor tissues from HPA database; (**B**) CCK8 assay was performed in NC group and T24 and UC3 cells transfected with si-SBF2-AS1. **C** Transwell assay was used to determine the migration and invasion ability of T24 and UC3 cells in NC group and transfected with si-SBF2-AS1. **D** Cell colony formation assay of NC group and T24 and UC3 cells transfected with si-SBF2-AS1

We verified the protein expression level of HNRNPA2B1 in normal tissue and tumor tissue by HPA database, the darker the color, the higher the expression in tumor tissue, suggesting that HNRNPA2B1 is highly expressed not only in normal tissue but also in tumor tissue, and the expression level is higher in tumor tissue (Fig. 9A). To investigate the role of SBF2-AS1 in bladder cancer cells, we designed siRNA to silence SBF2-AS1 expression in T24 and UC3 cells. Subsequently, CCK8, Transwell and colony formation assays were performed on T24 and UC3 cells transfected with si-SBF2-AS1. CCK8 results showed that the cell proliferation ability of NC group was significantly stronger than that of si-SBF2-AS1 group at 24, 48 and 72 h (Fig. 9B). Transwell results showed that knocking down SBF2-AS1 significantly limited the migration and invasion ability of T24 and UC3 cells (Fig. 9C). In addition, the colony formation assay of tumor cells showed that the proliferation ability of T24 and UC3 cells transfected with si-SBF2-AS1 was significantly lower than that of NC group (Fig. 9D). These results suggest that knocking down the expression of SBF2-AS1 can inhibit the proliferation, migration and invasion of bladder cancer cells.

## Discussion

Bladder cancer is the ninth most common malignant disease and the 13th most common cause of cancer death worldwide [21]. Environmental or occupational exposure to carcinogens, especially tobacco, is a major risk factor for bladder cancer [22]. At present, the clinical treatment of bladder cancer is mainly through surgical resection of solid tumor, followed by radiotherapy and chemotherapy, but some patients are in the middle and advanced stage of the disease at the time of diagnosis, and only a few patients with advanced tumor are qualified for surgical resection [2]. In recent years, the rise of immunotherapy has broken the stagnant state of bladder cancer research and treatment after intravesical instillation of BCG (BCG) in the treatment of local non-muscular invasive bladder cancer (NMIBC). At the same time, it will also rewrite the standard treatment mode of advanced bladder cancer [23]. However, while immunotherapy has made a major breakthrough in bladder cancer, it also faces great challenges, such as not all patients respond to immunotherapy and some patients develop acquired resistance after gaining initial benefits [24]. Biomarkers currently used to predict whether patients are responsive to immunotherapy include PD-L1 expression, luminal and basal typing of bladder cancer, tumor mutation load, microsatellite instability, etc. [25]. Therefore, future research should actively look for markers that can effectively predict the effect of immunotherapy, and explore immunotherapy strategies with higher specificity and fewer adverse reactions.

In this study, we used the Imvigor210 dataset, a study of patients with metastatic urothelial cancer treated with anti-PD-L1 drugs, to identify genes that were different between the anti-PD-L1 treatment response group and the non-response group and intersected with the lncRNA of bladder cancer in the TCGA cohort to obtain lncRNA associated with immunotherapy. We used multivariate Cox regression analysis to establish an immunotherapy-related lncRNA model, which can well predict the prognosis of patients, as well as predict the sensitivity of various chemotherapeutic drugs and the response to immunotherapy. The low-risk group has a significant effect on anti-PD-L1 immunotherapy, and the IPS score of the low-risk group is significantly higher than that of the high-risk group on whether or not to give anti-PD-1 and anti-CTLA4 immunotherapy, which suggests that the low-risk group has a better effect on immunotherapy. In this study, we also constructed a group of ceRNA networks, namely SBF2-AS1/has-miR-582-5p/HNRNPA2B1 regulatory axes, through immunotherapy-related lncRNA and mRNA.

According to the results of previous studies, SBF2-AS1 can be used as a molecular sponge of miRNA to promote the progression of many kinds of tumors, such as diffuse large B-cell lymphoma [26], hepatocellular carcinoma [27], non-small cell lung cancer [28], and cervical cancer [29]. Studies by Xia et al. have shown that SBF2-AS1 can promote the tumorigenesis and progression of breast cancer by counteracting its inhibitory effect on RRS1 by acting as a sponge of miR-143 [30]. Xu et al. found that miR-582-5p regulates the immune escape of non-small cell lung cancer cells by interacting with UCHL3 [31]. Another study found that miR-582-5p inhibits the occurrence and development of bladder cancer by inhibiting the expression of TTK [32]. Heterogeneous ribonucleoprotein A2B1 (HNRNPA2B1) can recognize viral DNA and promote the production of interferon α / β, thereby regulating cytoplasmic antiviral innate immunity [33]. Jiang et al. reported the role of HNRNPA2B1 in multiple myeloma and found that HNRNPA2B1 is an M6A reader, and its overexpression promotes the progression of multiple myeloma [34]. In addition, other studies have shown that HNRNPA2B1 can promote the progression of oral squamous cell carcinoma and gastric cancer [35, 36]. These results suggest that SBF2-AS1 and HNRNPA2B1 are oncogenes and has-miR-582-5p is a tumor suppressor gene. We infer that SBF2-AS1 regulates HNRNPA2B1 transcription and related signal pathways through has-miR-582-5p may affect the occurrence and development of bladder cancer and immune escape.

Previous studies have shown that overexpression of HNRNPA2B1 plays a role in endocrine resistance of cancer, enabling cancer cells to increase their vitality and obtain stem cell characteristics. Therefore, the treatment of targeted HNRNPA2B1 may be a therapeutic strategy for patients with endocrine resistant cancer [20]. Therefore, in our study, we found the first eight small molecules with the highest affinity by docking HNRNPA2B1 proteins with small molecular inhibitors, of which Ceftolozane has been reported to be used in clinical trials of hospital-acquired / ventilator-associated bacterial pneumonia (HABP/VABP) [37]. Saquinavir can be used to treat HIV-infected patients [38]. Our results show that these small molecules with high affinity to HNRNPA2B1 can play a role in endocrine resistance therapy of cancer by targeting HNRNPA2B1, but the specific mechanism and application value still need further mechanism exploration and clinical trials.

Our study has limitations. First, the analyses and conclusions of our study were based on public databases, which may have led to inherent case selection bias. In addition, although our results were validated on multiple external data sets, larger clinical cases are needed to further validate the accuracy of our results. The AUC value of our model is less than 0.7, and the prediction effect still has room for improvement. However, the AUC value of our model is higher than that of most clinical features, indicating that our model is superior to most clinical features in predicting the prognosis of patients, such as tumor stage, TNM stage and so on. Finally, further and more in-depth in vivo and in vitro experiments are needed to explore the function of immune-related lncRNA in bladder cancer.

## Conclusion

In summary, this study identified immunotherapy-related lncRNA in bladder cancer. We also established and verified a model based on immunotherapy-related lncRNA to predict the prognosis of patients with bladder cancer, and showed a good predictive ability. We further assessed the differences in immune microenvironment, chemotherapeutic drug sensitivity and immunotherapy response between high-risk and low-risk groups. Then we build a ceRNA network based on SBF2-AS1 and find small molecular drugs that can target HNRNPA2B1 through molecular docking. Finally, the role of SBF2-AS1 in bladder cancer was preliminarily verified by in vitro experiments. The above results are helpful for the identification of immunotherapy-related lncRNA and provide a new strategy for clinical immunotherapy and the development of new immunotherapy drugs.

## Supplementary Information

**Additional file 1: Table S1.** Univariate and multivariate COX results of 6 immunotherapy-related lncRNAs. **Table S2.** Results of has-miR-582-5p univariate and multivariate cox analysis. **Table S3.** Detailed relationship between HNRNPA2B1 and small molecular drugs. **Figure S1.** Prognostic analysis of has-miR-582-5p and the relationship between SBF2-AS1 and immune microenvironment.

## Data Availability

The study’s GEO database (GSE31684) can be downloaded from https://www.ncbi.nlm.nih.gov/gds/. The TCGA-BLCA gene matrix presented in the study could be obtained from the TCGA database(https://portal.gdc.cancer.gov/).

## Abbreviations

TCGA: The Cancer Genome Atlas
GEO: Gene Expression Omnibus
BC: Bladder Cancer
PD-1: Programmed cell death 1
PD-L1: Programmed cell death 1 ligand 1
CTLA4: Cytotoxic T-lymphocyte-associated protein 4
ICI: Immune checkpoint inhibitors
lncRNA: Long non-coding RNA
ceRNA: Competitive endogenous RNA
miRNA: microRNA
lncRNA SBF2-AS1: Long non-coding RNA SBF2 antisense RNA 1
HNRNPA2B1: Heterogeneous nuclear ribonucleoprotein A2B1
NMIBC: Non-muscle-invasive bladder cancer
TILs: Tumor-infiltrating lymphocytes
CDF: Cumulative distribution function
TME: Tumor microenvironment
OS: Overall survival
ROC: Receiver Operation Characteristic
NES: Normalized enrichment score
TMB: Tumor mutation burden
IC50: Half maximal inhibitory concentration
GDSC: Genomics of Drug Sensitivity in Cancer
PPI: Protein–protein interaction
KM: Kaplan–Meier
ssGSEA: Single-sample gene set enrichment analysis
TIDE: Tumor immune dysfunction and exclusion
GSEA: Gene set enrichment analysis
GO: Gene Ontology
KEGG: Kyoto Encyclopedia of Genes and Genomes
