# Supplementary material for: Identification of immunotherapy-related lncRNA signature for predicting prognosis, immunotherapy responses and drug candidates in bladder cancer
Source: BMC Cancer. 2023 Apr 18;23:355. doi: 10.1186/s12885-023-10828-z (PMC10111848; doi:10.1186/s12885-023-10828-z)
Supplement: Supplementary file 1 — Additional file 1: Table S1. Univariate and multivariate COX results of 6 immunotherapy-related lncRNAs. Table S2. Results of has-miR-582-5p univariate and multivariate cox analysis. Table S3. Detailed relationship between HNRNPA2B1 and small molecular drugs. Figure S1. Prognostic analysis of has-miR-582-5p and the relationship between SBF2-AS1 and immune microenvironment. [file 12885_2023_10828_MOESM1_ESM.docx]

**Table S1 Univariate and multivariate COX results of 6 immunotherapy-related lncRNAs**

|  | Univariate analysis | | Multivariate analysis | |
| --- | --- | --- | --- | --- |
| gene | HR(95% CI) | P value | HR(95% CI) | P value |
| TFAP2A-AS1 | 0.695 (0.509 - 0.950) | 0.022 | 0.787 (0.656 - 0.943) | 0.01 |
| SBF2-AS1 | 1.517 (1.028 - 2.238) | 0.036 | 1.202 (1.000 - 1.445) | 0.05 |
| HHLA3 | 1.069 (0.877 - 1.303) | 0.51 | 1.004 (0.802 - 1.257) | 0.972 |
| MIRLET7BHG | 1.005 (0.735 - 1.374) | 0.977 | 1.126 (0.783 - 1.621) | 0.522 |
| FLG-AS1 | 2.056 (0.877 - 4.817) | 0.097 | 1.653 (0.653 - 4.186) | 0.289 |
| RRN3P2 | 0.159 (0.040 - 0.632) | 0.009 | 0.459 (0.248 - 0.850) | 0.013 |

**Table S2 Results of has-miR-582-5p univariate and multivariate cox analysis**

| Characteristics | Total(N) | Univariate analysis | |  | Multivariate analysis | |
| --- | --- | --- | --- | --- | --- | --- |
|  |  | Hazard ratio (95% CI) | P value |  | Hazard ratio (95% CI) | P value |
| Pathologic T stage | 383 |  | **< 0.001** |  |  |  |
| T1&T2 | 126 | Reference |  |  | Reference |  |
| T3&T4 | 257 | 2.256 (1.554 - 3.274) | **< 0.001** |  | 1.743 (1.155 - 2.629) | **0.008** |
| Pathologic N stage | 373 |  | **< 0.001** |  |  |  |
| N0 | 240 | Reference |  |  | Reference |  |
| N1&N2&N3 | 133 | 2.336 (1.714 - 3.184) | **< 0.001** |  | 2.024 (1.462 - 2.801) | **< 0.001** |
| hsa-miR-582-5p | 417 | 0.900 (0.788 - 1.029) | 0.123 |  | 0.968 (0.835 - 1.122) | 0.666 |

**Table S3** **Detailed relationship between HNRNPA2B1 and small molecular drugs**

**HNRNPA2B1**

**A：7WM3: ZINC000004097343（Itc）**

**Ligand Receptor Interaction Distance E (kcal/mol)**

**5-ring NH1 ARG 153 (A) pi-cation 4.02 -6.1**

**6-ring CB ARG 185 (A) pi-H 4.41 -0.5**

**B：7WM3: ZINC000095564694（Naloxegol）**

**Ligand Receptor Interaction Distance E (kcal/mol)**

**O41 41 NZ LYS 17 (A) H-acceptor 2.99 -5.4**

**C：7WM3: ZINC000003978005（Dihydroergotamine）**

**Ligand Receptor Interaction Distance E (kcal/mol)**

**C4 4 O VAL 170 (A) H-donor 3.21 -0.6**

**5-ring CB ALA 98 (A) pi-H 3.77 -0.9**

**D：7WM3: ZINC000169289767（Trypan Blue）**

**Ligand Receptor Interaction Distance E (kcal/mol)**

**N37 37 O GLY 106 (A) H-donor 3.07 -1.3**

**O20 20 NH1 ARG 190 (A) H-acceptor 2.94 -5.4**

**O20 20 NH2 ARG 190 (A) H-acceptor 3.38 -0.5**

**O21 21 NH1 ARG 153 (A) H-acceptor 3.34 -0.7**

**O21 21 NH2 ARG 153 (A) H-acceptor 2.95 -3.9**

**O22 22 NH2 ARG 190 (A) H-acceptor 3.01 -3.5**

**O28 28 NH1 ARG 185 (A) H-acceptor 2.93 -2.6**

**O30 30 NH1 ARG 185 (A) H-acceptor 3.01 -4.3**

**O41 41 N LYS 186 (A) H-acceptor 3.03 -2.2**

**O48 48 NZ LYS 173 (A) H-acceptor 3.04 -7.7**

**O49 49 NZ LYS 173 (A) H-acceptor 2.79 -0.8**

**O50 50 NZ LYS 104 (A) H-acceptor 2.77 -11.4**

**O56 56 CE LYS 186 (A) H-acceptor 3.44 -0.6**

**O20 20 NH1 ARG 190 (A) Ionic 2.94 -4.9**

**O20 20 NH2 ARG 190 (A) Ionic 3.38 -2.4**

**O21 21 NH1 ARG 153 (A) Ionic 3.34 -2.6**

**O21 21 NH2 ARG 153 (A) Ionic 2.95 -4.8**

**O22 22 NH1 ARG 190 (A) Ionic 3.97 -0.6**

**O22 22 NH2 ARG 190 (A) Ionic 3.01 -4.4**

**O28 28 NH1 ARG 185 (A) Ionic 2.93 -4.9**

**O28 28 NH2 ARG 185 (A) Ionic 3.75 -1.1**

**O30 30 NH1 ARG 185 (A) Ionic 3.01 -4.4**

**O30 30 NH2 ARG 185 (A) Ionic 3.63 -1.4**

**O48 48 NZ LYS 173 (A) Ionic 3.04 -4.2**

**O49 49 NZ LYS 173 (A) Ionic 2.79 -6.1**

**O50 50 NZ LYS 104 (A) Ionic 2.77 -6.2**

**O56 56 NZ LYS 186 (A) Ionic 3.79 -1.0**

**E：7WM3: ZINC000022448097（Eovist）**

**Ligand Receptor Interaction Distance E (kcal/mol)**

**C11 11 OE2 GLU 101 (A) H-donor 2.94 -1.6**

**C14 14 OE2 GLU 101 (A) H-donor 3.04 -0.5**

**C25 25 OE1 GLU 101 (A) H-donor 3.24 -1.0**

**O19 19 CA ALA 107 (A) H-acceptor 3.27 -0.7**

**O20 20 CE LYS 186 (A) H-acceptor 2.99 -0.6**

**O24 24 N ALA 98 (A) H-acceptor 3.09 -5.1**

**O32 32 NZ LYS 104 (A) H-acceptor 3.37 -4.1**

**N12 12 OE2 GLU 101 (A) Ionic 3.33 -2.6**

**O20 20 NZ LYS 186 (A) Ionic 3.67 -1.3**

**O28 28 NZ LYS 104 (A) Ionic 3.09 -3.9**

**O32 32 NZ LYS 104 (A) Ionic 3.37 -2.4**

**O33 33 NZ LYS 173 (A) Ionic 2.99 -4.6**

**6-ring CB ARG 185 (A) pi-H 3.84 -0.7**

**F：7WM3: ZINC000009164421（Ceftolozane）**

**Ligand Receptor Interaction Distance E (kcal/mol)**

**C18 18 O LYS 186 (A) H-donor 3.23 -0.6**

**O39 39 NZ LYS 173 (A) H-acceptor 2.92 -3.2**

**O40 40 NZ LYS 104 (A) H-acceptor 3.15 -0.9**

**O39 39 NZ LYS 173 (A) Ionic 2.92 -5.0**

**O40 40 NZ LYS 104 (A) Ionic 3.15 -3.6**

**O40 40 NZ LYS 173 (A) Ionic 3.86 -0.8**

**5-ring CA LEU 171 (A) pi-H 4.53 -1.5**

**G：7WM3: ZINC000026985532（Sqv）**

**Ligand Receptor Interaction Distance E (kcal/mol)**

**O24 24 CE LYS 173 (A) H-acceptor 3.12 -0.7**

**6-ring NZ LYS 173 (A) pi-cation 3.45 -0.9**

**H：7WM3: ZINC000028467879（Ceftriaxone）**

**Ligand Receptor Interaction Distance E (kcal/mol)**

**N37 38 OD1 ASP 167 (A) H-donor 2.84 -0.9**

**O15 15 NZ LYS 104 (A) H-acceptor 2.78 -4.9**

**O16 16 NZ LYS 173 (A) H-acceptor 2.98 -2.9**

**S30 31 N LYS 186 (A) H-acceptor 4.43 -0.9**

**O15 15 NZ LYS 104 (A) Ionic 2.78 -6.1**

**O16 16 NZ LYS 173 (A) Ionic 2.98 -4.6**





**Figure S1.** **Prognostic analysis of has-miR-582-5p and the relationship between SBF2-AS1 and immune microenvironment.** (A) Kaplan-Meier curve of high / low has-miR-582-5p expression group in bladder cancer. (B) The difference of SBF2-AS1 and HNRNPA2B1 expression between normal bladder tissue and tumor tissue in TCGA data.(C) The difference of has-miR-582-5p expression between normal bladder tissue and tumor tissue in TCGA data.(D) Difference of SBF2-AS1 expression between anti-PD-L1 response group and non-response group. Differences in Immunescore(E) and Stromalscore(F) between high and low SBF2-AS1 expression groups. Differences in Tumorpuity(G)、 Immunescore(H) and Stromalscore(I) between high and low hsa-miR-582-5p expression groups.
